# Precision agriculture management based on a surrogate model assisted multiobjective algorithmic framework

**DOI:** 10.1038/s41598-023-27990-w

**Published:** 2023-01-20

**Authors:** Du Cheng, Yifei Yao, Renyun Liu, Xiaoning Li, Boxu Guan, Fanhua Yu

**Affiliations:** 1 Department of Computer Science, Changchun Normal University, Changchun, Jilin Province China; 2 Department of Computer Science, Guangxi Normal University, Guilin, Guangxi Province China; 3 Department of Computer Science, Beihua University, Jilin City, Jilin Province China

**Keywords:** Ecology, Hydrology

## Abstract

Sustainable intensification needs to optimize irrigation and fertilization strategies while increasing crop yield. To enable more precision and effective agricultural management, a bi-level screening and bi-level optimization framework is proposed. Irrigation and fertilization dates are obtained by upper-level screening and upper-level optimization. Subsequently, due to the complexity of the problem, the lower-level optimization uses a data-driven evolutionary algorithm, which combines the fast non-dominated sorting genetic algorithm (NSGA-II), surrogate-assisted model of radial basis function and Decision Support System for Agrotechnology Transfer to handle the expensive objective problem and produce a set of optimal solutions representing a trade-off between conflicting objectives. Then, the lower-level screening quickly finds better irrigation and fertilization strategies among thousands of solutions. Finally, the experiment produces a better irrigation and fertilization strategy, with water consumption reduced by 44%, nitrogen application reduced by 37%, and economic benefits increased by 7 to 8%.

## Introduction

One of the major challenges facing humanity in the twenty-first century is the way to meet the growing demand for food while reducing waste of resources^1^. A study shows that the global demand for food is expected to grow by between 60 and 110 percent between 2005 and 2050^2^, which means that it is increasingly important to implement precision crop management. In addition, in arid and semi-arid regions, the use of irrigation water plays a vital role in agricultural production. Globally, irrigation accounts for 85% of the total water used in agriculture and generates about 40% of the total food production^3^. At the same time, fertilizer application is another important factor in ensuring increased crop yield. Soil hardening and environmental pollution caused by fertilizer application and the shortage of water resources are universally concerned in the world. In this regard, the optimization of irrigation and fertilizer strategies can not only improve the crop yield but also reduce production costs, protect the environment and save resources^4^. For example, in Florida, USA^5^, the optimization of irrigation and fertilizer strategies can reduce irrigation by 48% and fertilizer application by 26% with the same yield.

Recent studies have shown that irrigation and fertilizer application should be managed simultaneously^4,6^. Excessive fertilizer application can inhibit plant growth and even lead to higher mortality of seedlings in the drought environment. Effective water management facilitates the incorporation of nutrients into the soil. Therefore, the optimization of irrigation and fertilizer strategies plays a key role in improving crop yield in areas where both nutrients and water are limited.

Over the past 40 years, many countries around the world have been actively developing crop models that have so far undergone a process of development from infancy to maturity, from theory to practice. DSSAT (DSSAT v4.5, Joint development by international organizations, USA) is one of the most widely used model series, which has been developed different models for different crops^7^. DSSAT currently consists of 26 different crop simulation models, such as CERES^8^, CROPGRO^9^, GANEGRO, PNUTGR, etc. These models have been widely used and improved in the past.

Many real-world optimization problems often have multiple conflicting objectives to be optimized. Such problems are called multi-objective optimization problems (MOPs). MOPs are widely used to solve the problems of irrational organization and waste of resources in modern agricultural industry. Y. W. Zhou and Fan^10^ have optimized the structure of the agricultural industry through MOP based on genetic algorithm for achieving sustainable development. Ragkos and Psychoudakis^11^ applied the weighted goal programming approach to analyze the impact on the crop economy by reducing the use of irrigation and chemicals, and thus selected the options that will provide good management of agriculture in the future. Sarker and Ray^12^ proposed a variant of NSGA-II being applied to optimize crop planning practices. The program examined an agricultural area containing multiple farms and sought the best solution for distributing crops on the farms. It attempted to minimize investment and maximize profit. Han, Hu, Mao and Wan^13^ pooled a specific set of agricultural production areas into a specified number of service areas while assigning a maintenance service facility to each area, and used MOP to minimize the total mileage between the demand point and the facility while the service area is overloaded with demand.

A lot of multi-objective evolutionary algorithms (MOEAs) were proposed in the past decades due to the ability to handle different kinds of decision variables, e.g., the elitist non-dominated sorting genetic algorithm (NSGA-II)^14^, the region-based selection algorithm (PESA-II)^15^, the multi-objective evolutionary algorithm based on decomposition (MOEA/D)^16^, and the improved strength Pareto evolutionary algorithm (SPEA2)^17^. These MOEAs have been shown to be effective with two or three objectives^18^. Despite these advantages, these MOEAs are often criticized due to slow convergence and the large number of function evaluations required before an acceptable solution can be found.

Since traditional evolutionary algorithms (EAs) rely heavily on fitness evaluations (FEs) to assist the evolutionary process^19^, they often lead to deterioration of algorithm performance when the number of FEs is insufficient. In many real-world problems, FEs may be too expensive to obtain^20^. In contrast, by using data and surrogate models to reduce FEs and assist the evolutionary process, data-driven evolutionary algorithms(DDEAs)are able to obtain satisfactory solutions within a limited number of available FEs^21–23^, for example, Chen and Luo^24^ showed the DDEA is very promising for a well-placement optimization problem of two-dimensional reservoir and joint optimization of Egg model, Long & Li^25^ showed the super-performance of DDEA in complex situations in terms of power output and execution time, Belhaiza et al.^26^ optimized the vehicle routing problem with multiple time Windows by the DDEA method. Since DDEAs rely heavily on surrogate models, a proper surrogate model is critical to the performance of DDEAs. Choosing the right model and method to build surrogates can improve the performance of DDEAs, such as Polynomial regression^27^, Kriging interpolation^28^, Radial Basis Function interpolation^29,30^, Neural Networks^31,32^, and Support Vector Machine regression^33^. In addition, many authors have proposed a collection of various types of surrogate models that are used to improve^34,35^ the predictive power of the overall approximation model.

In recent years, there have been various frameworks that apply multi-objective optimization algorithms to agriculture. In traditional bi-level optimization frameworks^36^, not only is the selection of dates not reprocessed but the large number of strategies generated by multiple objectives are not filtered accordingly. This not only increases the code running time significantly, but also the thousands of strategies are not decision-maker friendly. Therefore, in this paper, in order to achieve more precision and effective agricultural management, the bi-level screening and bi-level optimization (BSBOP) framework is proposed to solve such problems. The crop growth in the experiment is simulated by DSSAT and the management of irrigation and fertilizer application during the crop growth cycle is optimized by MOP. Since MOP generates a large number of solutions, decision-makers can choose the appropriate solution for agricultural management according to local conditions and their preferences. In addition, DSSAT consumes a certain amount of time for each run, which becomes unacceptable to decision-makers as the problem becomes more complex. So, the combination of DDEA and DSSAT in the lower-level optimization (LOP) goes to reduce the time consumption. The main contributions of this paper as follows.

- The experiment produces a better irrigation and fertilization strategy, with water consumption reduced by 44%, nitrogen application reduced by 37%, and economic benefits increased by 7 to 8%.
- To achieve more precision and effective agricultural management, BSBOP framework is proposed, which makes the algorithm and DSSAT model more tightly integrated and not only reduces the program running time, but also is more friendly to decision-makers.
- Applying DDEAs to DSSAT makes it possible to solve the severely time-consuming problem with limited computational budgets. Relevant strategies are adopted to ensure the convergence and diversity of the algorithm.

## Materials and methods

### Study area

**Figure 1:**
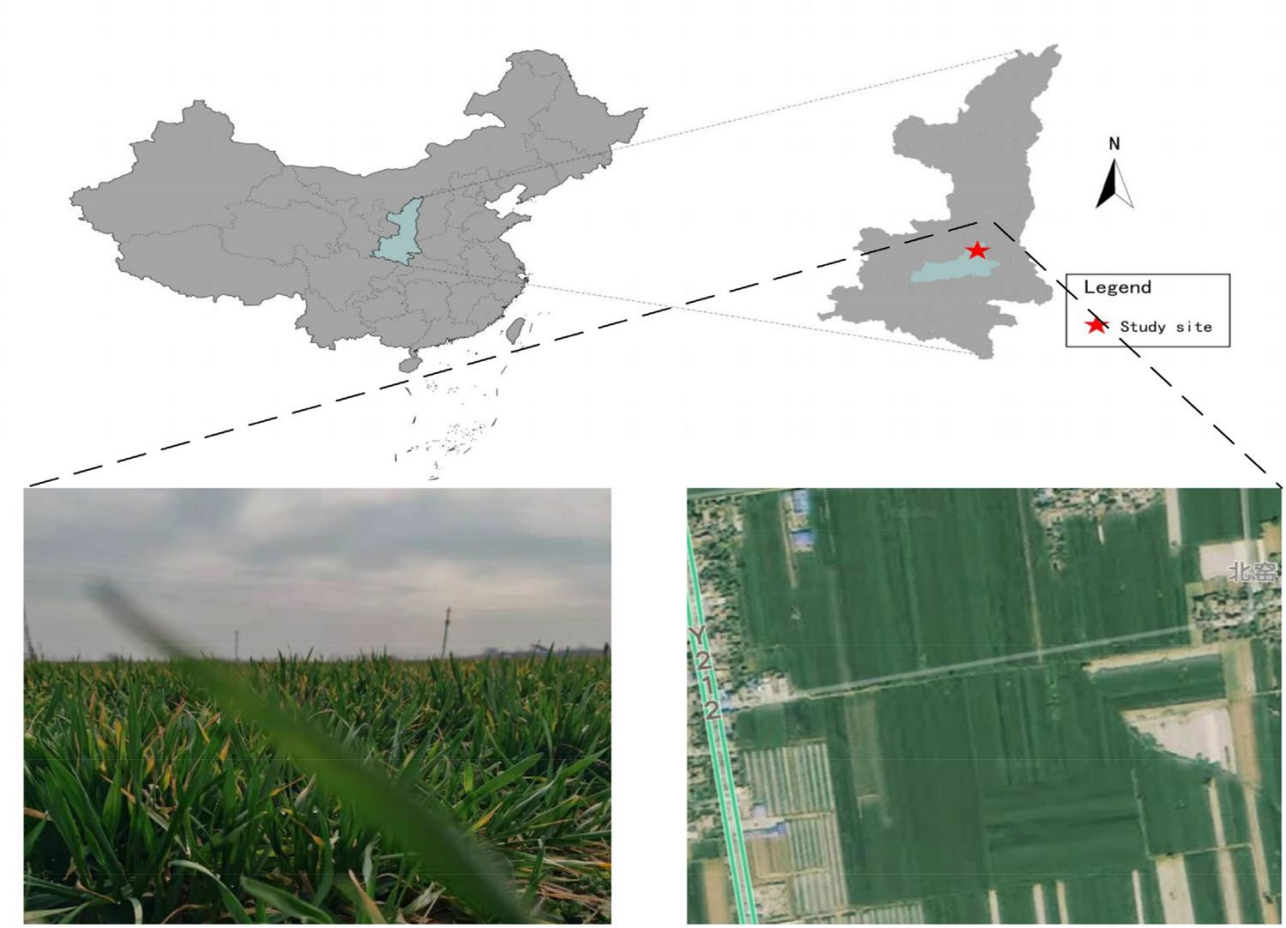
Location of the field of study (The satellite imagery supporting this study was obtained using Baidu Maps (Android version—16.4.0.1195). The URL is (https://map.baidu.com/@14256795.568410998,5210675.606268121,8.67z.).

The study area is located in Lintong District, Xi'an City, Shaanxi Province, China (34° 21′ 59.94″, 109° 12′ 51.012″) (Meteorologists, 2020b). The study area is located in northwestern China (Fig. 1), which is a Warm temperate semi-humid continental climate with distinct cold, warm, dry and wet seasons. Winter is cold, windy, foggy, and with little rain or snow. Spring is warm, dry, windy, and variable. The summer is hot and rainy, with prominent droughts and thunderstorms, and high wind. Autumn is cool, the temperature drops rapidly and autumn showers are obvious. The annual average temperature is 13.0–13.7 °C, the coldest January average temperature is −1.2–0 °C, the hottest July average temperature is 26.3–26.6 °C, the annual extreme minimum temperature is −21.2 °C, Lantian December 28, 1991, the annual extreme maximum temperature is 43.4 °C, Chang'an June 19, 1966. Annual precipitation is 522.4–719.5 mm, increasing from north to south. July and September are the two obvious peak precipitation months. The annual sunshine hours range from 1646.1 to 2114.9 h. The dominant wind direction varies from place to place, with the northeast wind in Xi'an, west wind in Zhouzhi and Huxian, east-northeast wind in Gaoling and Lintong, southeast wind in Chang'an, and northwest wind in Lantian. Meteorological disasters include drought, continuous rain, heavy rain, flooding, urban flooding, hail, gale, dry hot wind, high temperature, lightning, sand and dust, fog, haze, cold wave, and low-temperature freeze.

Wheat (XiNong 805) was planted on September 24, 2019 and matured for harvest on May 28, 2020 (We warrant that we have the right to collect and manage wheat (XiNong 805). In addition, the study is in compliance with relevant institutional, national, and international guidelines.). Among the six strategies in the experiment (Table 1), we focused on strategies 1 and 4, fixed irrigation dates optimization and fixed fertilizer application dates optimization. Based on the custom of the study area, three days of diffuse irrigation were selected for Strategy 1. Three days of fertilization of the urea and three days of irrigation were selected for Strategy 4. The best practice for Strategy 1 was total irrigation of 201 mm for the total season and a total of 7388 kg/ha of wheat was obtained for this simulation, while the best practice for Strategy 4 was total irrigation of 197 mm for the total season and a total fertilizer application of 282 kg/ha for the total season. A total of 7894 kg/ha of wheat was obtained for this simulation.

**Table 1:** Details of the 6 strategies of the experimental setup.

Strategy No	Optimization type	Application dates	Objectives
1	Fixed irrigation dates	3 fixed irrigation dates (dates from the field experiment)	Max: Yield Min: Irrigation applied
2	Optimal irrigation dates	3 variable irrigation dates	Max: Yield Min: Irrigation applied
3	Optimal irrigation dates based on surrogate model	3 variable irrigation dates	Max: Yield Min: Irrigation applied
4	Fixed fertilizer application date	3 fixed irrigation dates (dates from the Strategy 2) 3 fixed nitrogen dates (dates from the field experiment)	Max: Yield Min: Irrigation applied Min: Nitrogen applied
5	Optimal fertilizer application date	3 fixed irrigation dates (dates from the Strategy 2 3 variable nitrogen dates	Max: Yield Min: Irrigation applied Min: Nitrogen applied
6	Optimal fertilizer application date based on surrogate model	3 fixed irrigation dates (dates from the Strategy 2) 3 variable nitrogen dates	Max: Yield Min: Irrigation applied Min: Nitrogen applied

### DSSAT model

DSSAT, one of the most widely used crop growth models, is an integrated computer system developed by the University of Hawaii under the authority of the U.S. Agency for International Development (USAID). It aims to aggregate various crop models and standardize the format of model input and output variables to facilitate the diffusion and application of models^7^, thereby accelerating the diffusion of agricultural technology and providing decision making and countermeasures for the rational and efficient use of natural resources in developing countries.

The DSSAT 4.5 model integrates all crop models into the simulation pathway-based CSM (Cropping System Model) farming system model, which uses a set of simulated soil moisture, nitrogen, and carbon dynamics codes, while crop growth and development are stimulated through the CERES^37,38^, CROPGRO^39^, CROPSIM, and SUBSOR modules. DSSAT is applicable to single sites or same type zones and can be extrapolated to the regional level through Geographic Information System (GIS).

DSSAT–CSM simulates the growth process of crops grown on a uniform land area under prescribed or simulated management^40^, and the changes in soil water, carbon and nitrogen with under tillage systems. The DSSAT model is a decision support system supported by crop simulation models, which, in addition to data support, provides methods for calculating and solving problems, and provides decision-maker with the results of their decisions. It also provides scientific decisions for farmers to provide different cultivation management measures (e.g., proper fertilization and irrigation for crops) in different climatic years.

### Inputs and outputs of the model

The DSSAT model has four main user-editable input files and various output files. The input files include crop management^7,41^, soil, weather, and cultivar parameter files; the output files include three types: (1) output files, (2) seasonal output files, and (3) diagnostic and management files.

Crop management data: Crop management data provides basic information about crop growth. Detailed and accurate parameter provision is the basis for improving the accuracy of model simulation. Crop management parameters include crop variety, soil type, meteorological name, previous season crop, sowing period, sowing density, sowing depth, irrigation amount and time, fertilizer application amount and time, the initial condition of the soil, pest management, tillage frequency and method, etc. Some of these parameters are not easily available in field experiments and can be obtained from other test sites or from existing documentation. On the other hand, if there are missing values in the model, it will increase the simulation error of the model (this situation is hard to avoid). Therefore, in this study, the parameters were selected based on the principle of being both detailed and easily available.

*Soil data* Soil data contains various parameters of the soil section plane, including soil color, soil slope, soil capacity, organic carbon, soil nitrogen content, drainage properties, the proportion of clay, particles, and stones in the soil. Similar to the governing documents, the more complete the parameters the smaller the error value of the simulation. The various physical and chemical properties of the soil for this study were obtained from the China Soil Database at the time of the study. The various physical and chemical properties of the soil for this study were obtained from the China Soil Database.

**Figure 2:**
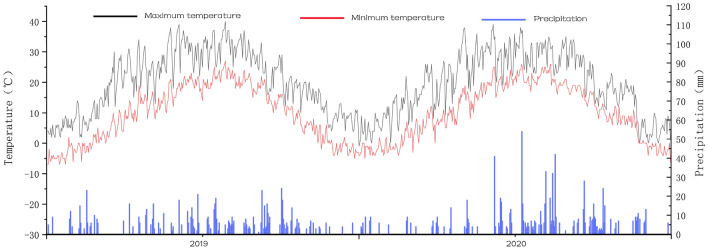
Precipitation and maximum and minimum temperatures during 2019–2020.

*Weather data* The DSSAT model uses daily weather data as weather input data for the model. The model requires a minimum of four daily weather data in order to accurately simulate the water cycle in soil plants (Fig. 2). These are:(1) daily solar radiation energy (MJM); (2) daily maximum temperature (°C); (3) daily minimum temperature (°C); and (4) daily precipitation (mm). Weather data were obtained from the China Meteorological Administration. Weather data were obtained from the China Meteorological Administration.

*Model calibration* Adjusting the cultivar parameter is very important to accurately simulate the local growing environment. In this experiment, we collected field data for 2019 and 2020, and adjusted the parameters in the cultivar parameter files by trial-and-error method to make the simulation process more closely match the actual local crop growth process.

### Multi-objective optimization algorithm

Multi-objective optimization techniques have been successfully applied in many real-world problems. In general^42–44^, MOPs produce a set of optimal solutions that together represent a trade-off between conflicting objectives, and such solutions are called Pareto optimal solutions (PS). These PS cannot make any solution better without compromising the other solutions. Therefore, when solving multi-objective problems, more PS are needed to find. Some MOPs aim to find all PS or at least a representative subset of them.

A multi-objective optimization problem can be stated as follows:

1 $$\mathrm{min }F\left(x\right)={({f}_{1}\left(x\right),\dots ,{f}_{k}(x))}^{T}$$

2 $$\mathrm{subject\;to}\;x\in \Omega$$

where $\Omega$ is the decision space,$F:\Omega \to {R}^{k}$ consists of $k$ real-value objective functions and ${R}^{k}$ is called the objective space. The attainable objective set is defined as the set $\{F(x)\in \Omega \}$.

### NSGA-II optimizer

We use non-dominated sorting genetic algorithm (NSGA-II) for Multiobjective optimization in R language. The NSGA-II algorithm is a classical multi-objective evolutionary algorithm with remarkable results in solving 2-objective and 3-objective problems^45^. It maintains the convergence speed and diversity of solutions by fast non-dominated sorting and crowding distance, selects the next population by elite selection strategy.

### Objective function

The multi-objective optimization problem varies one or more variables to maximize or minimize two or more objective problems. In the case of crop production, where decision-makers change irrigation and fertilizer application to maximize benefits, this study focuses on when to apply irrigation or fertilizer on the field and how much irrigation or nitrogen fertilizer to apply.

There are many crop models available that can be used as optimization objective functions, and DSSAT is definitely the best choice because it is easy to use and well-proven^36^. The user runs the model by entering defined soil, weather, variety, and crop management files, which are fed into the core of the model, the Crop Simulation Model (CSM). The model simulates the growth, development, and yield of crops grown on a uniform land area under management, as well as changes in soil water, carbon, and nitrogen over time under cropping systems. The CSM itself is a highly modular model system consisting of a number of sub-modules. Researchers have validated the output of these sub-modules as a whole under various crops, climate, and soil conditions.

Using DSSAT, it is easy to design a set of objective functions and optimize them, as in our case.

3 $$\mathrm{Max}:Y=\mathrm{DSSAT}\left.\left( {i}_{a0},\dots ,{i}_{\mathrm{aj}},{f}_{\mathrm{a}0},\dots ,{f}_{\mathrm{ad}},{D}_{i}\right.\right)$$

4 $$\mathrm{Min}:I=\sum_{n=0}^{j}{i}_{an}$$

5 $$\mathrm{Min}:F=\sum_{m=0}^{d}{f}_{am}$$

where $Y$ is yield,$I$ is the total amount of irrigation, $F$ is the total amount of nitrogen application, ${i}_{an}$ is the amount of irrigation at one time, ${f}_{am}$ is the amount of nitrogen applied at one time, $j$ is a number of applications of irrigation, and $d$ is a number of nitrogen applications. ${D}_{i}$ is a random date combination of irrigation time and fertilizer application time.

All other variables (e.g., climate, soil, location, crop variety) are kept constant during the optimization process. The irrigation unit is mm and the nitrogen application unit is kg/ha, the irrigation and nitrogen application amounts are positive integers by default (integer arithmetic reduces the program running time).

### Data-driven evolutionary algorithms

In general, the key to DDEAs is to reduce the required FEs and assist evolution through data. The data is generally utilized through surrogate model. The use of suitable surrogate model can be used in place of real FEs^46^. Thus, DDEAs have more advantages over EAs in solving expensive problems.

In terms of algorithmic framework, DDEAs contain two parts: surrogate model management (SMM) and evolutionary optimization part (EOP)^47,48^. The SMM part is used in order to obtain better approximations, while EOPs will use surrogate models in EAs to assist evolution. DDEAs can be divided into two types: online DDEAs and offline DDEAs^23^. Online DDEAs can be evaluated by real FEs with more new data. This new information can provide SMM with more information and construct a more accurate surrogate model^49^. Since DSSAT can obtain new data through FEs during the EOP process, the method used in this paper is online DDEAs. In contrast, offline DDEAs can only drive evolution through historical data.

Radial Basis Function (RBF) network is a single hidden layer feedforward neural network that uses a radial basis function as the activation function for the hidden layer neurons, while the output layer is a linear combination of the outputs of the hidden layer neurons. RBF was used to approximate each objective function. According to the investigation of multi-objective optimization problems with high computational cost, radial basis functions are often used as the surrogate model, mainly because RBF networks can approximate arbitrary nonlinear functions with arbitrary accuracy and have global approximation capability, which fundamentally solves the local optimum problem of BP networks, and the topology is compact, the structural parameters can be learned separately, and the convergence speed is fast.

In this paper, a new data-driven approach is proposed and place it in the lower-level optimization of the framework. RBF is utilized as the surrogate model and NSGA-II as the optimizer. Details are described in Algorithm 1.

### Data-driven method details


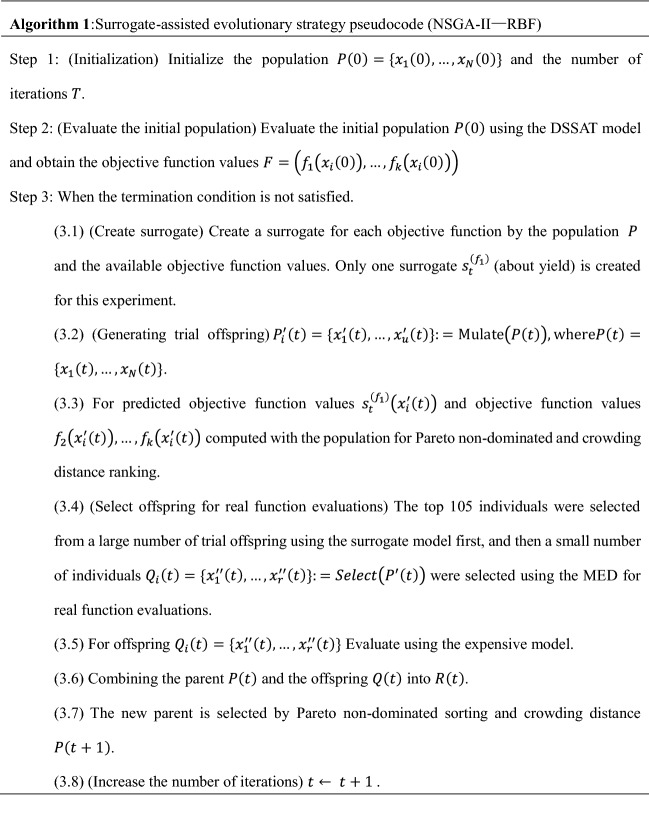


In step 1, the initial parent population is generated by randomly selecting points and the size is $N$. In step 2, we run DSSAT $N$ times to determine the objective function values of the $N$ initial population solutions. Next, the algorithm then loops through the generations. At the beginning of each loop, surrogate models, which one objective train one surrogate and denoted by ${s}_{t}^{\left({f}_{1}\right)}$ , were trained by the already obtained objective function values (step 3.1). The trial offspring ${P}_{i}^{^{\prime}}\left(t\right)=\{ {x}_{1}^{^{\prime}}\left(t\right),\dots ,{x}_{u}^{^{\prime}}\left(t\right)\}$ are generated by SBX and PM (step 3.2), then the trained surrogate model is used to predict the objective function values of trial offspring (step 3.3). The predicted objective function values are sorting by Pareto non-dominated and crowding distance (step 3.4), then $r$ offspring $Q_{i} \left( t \right) = \left\{ {x^{\prime\prime}_{1} \left( t \right), \ldots ,x^{\prime\prime}_{r} \left( t \right)} \right\}$ are selected from the trial offspring (step 3.5).The offspring are evaluated by the DSSAT (step 3.6), and after combining the parent population and offspring population (step 3.7), the new parent population are selected by Pareto non-dominated and crowding distance sorting (step 3.8).

### Maximum extension distance

MED guides a small number of individuals to approximate the entire PF. MED is defined as follow:

6 $$\mathrm{MED}\left({P}_{t}^{\left(q\right)}\right)=\mathrm{ND}\left({P}_{t}^{\left(q\right)}\right)\times \mathrm{TD}\left({P}_{t}^{\left(q\right)}\right)$$

where

$$\mathrm{ND}\left({P}_{t}^{\left(q\right)}\right)=\underset{z,q\ne z}{\mathrm{min}}\sum_{m=1}^{M}\left|{f}_{m}^{\left(q\right)}-{f}_{m}^{\left(z\right)}\right|$$

$$\mathrm{TD}\left({P}_{t}^{\left(q\right)}\right)=\sum_{z=1}^{P}\sum_{m=1}^{M}\left|{f}_{m}^{\left(q\right)}-{f}_{m}^{\left(z\right)}\right|$$

${P}_{t}^{\left(q\right)}$ is the *q*th individual in population *P*_*t*_ at the *t*th generation. $\mathrm{ND}\left({P}_{t}^{\left(q\right)}\right)$ calculates the minimum distance between ${P}_{t}^{\left(q\right)}$ and ${P}_{t}^{\left(z\right)}$. The larger $\mathrm{ND}\left({P}_{t}^{\left(q\right)}\right)$ value means a better individual diversity. $\mathrm{TD}\left({P}_{t}^{\left(q\right)}\right)$ calculates the summation of distance between ${P}_{t}^{\left(q\right)}$ and ${P}_{t}^{\left(z\right)}$. The larger $\mathrm{TD}\left({P}_{t}^{\left(q\right)}\right)$ value means that the solution ${P}_{t}^{\left(q\right)}$ has moved away from other individuals. A larger MED value means that an individual extends the overall boundary and an individual acquires better diversity.

### Modeling process

**Figure 3:**
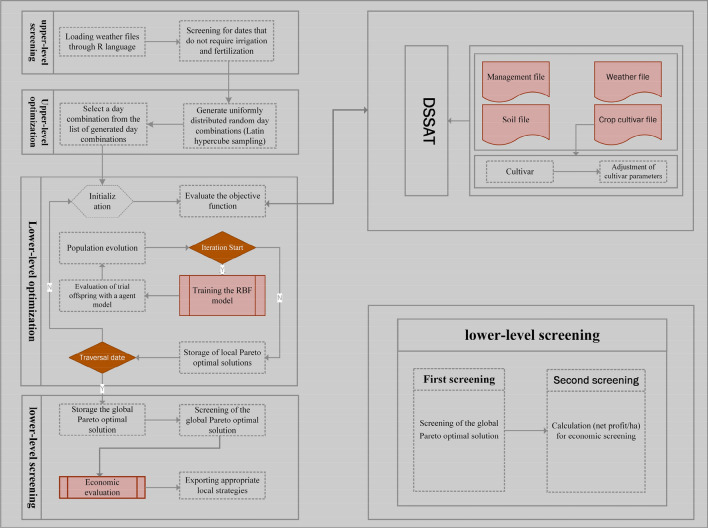
Proposed integrated bi-level screening, bi-level optimization and DSSAT framework.

To maximize crop yield and optimize the use efficiency of water and fertilizer in a given environment, BSBOP framework is proposed. Crop growth is simulated by DSSAT, the data-driven approach reduces the runtime of the overall framework while finding optimal management strategies. The overall framework includes four main parts: upper-level screening, upper-level optimization, lower-level optimization and lower-level screening (Fig. 3).

*Upper-level screening* The weather file in DSSAT was loaded by R language. The data are pre-processed with precipitation and solar radiation information to narrow down the date range for irrigation and fertilizer application. In other words, the date ranges for selecting irrigation and fertilization are restricted by the ULS.

*Upper-level optimization* Generating random combinations of dates by the Latin hypercube sampling method (LHS). The upper-level screening starts with referencing the two variables (number of irrigation and nutrient application events). LHS uses these variables to generate a series of uniformly distributed random day combinations. For example, date combinations generated by the LHS could be May 15, July 18 and August 1 for irrigation and May 30, June 30 and July 18 for nutrient application. From the series of uniformly distributed random day combinations, one will be selected and incorporated into the lower-level optimization.

*Lower-level optimization* The agricultural management strategy is optimized by the online data-driven approach proposed in Algorithm 1. Assuming three irrigation and three nitrogen application events are given, these events will be incorporated into the LOP, which consists of the RBF and NSGA-II. The population size of this paper is 105. The number of iterations varies according to the different strategies, and the objective function values are calculated by DSSAT. The main idea of applying Evolutionary multi-objective algorithms(EMO) and RBF to DSSAT is to generate a large number of trial offspring by traditional Simulated Binary Crossover (SBX) and Polynomial Mutation (PM), and then evaluate them using the trained surrogate model^50^. The objective values of the evaluation were then ranked by Pareto non-dominated and crowding distance, and the top 105 individuals were selected from a large number of trial offspring, after which a small number of individuals out of 105 were selected by Maximum Extension Distance (MED) for real function evaluation, and then combine the parents and offspring to select the next generation of parents by Pareto non-dominated and crowding distance sorting. Furthermore, in the numerical experiments, to ensure the superiority of the algorithm and reduce the experimental complexity, we use a relatively simple radial basis function (RBF) surrogate. The NSGA-II algorithm can be used for both bi-objective and tri-objective problems, so it can optimize the system by starting with the most critical objective and then adding additional objectives. For each solution in the population, the objective functions (1: maximize yield, 2: minimize irrigation application, 3: minimize nitrogen fertilizer application) will be evaluated by invoking the DSSAT model for these dates and the amount of fertilizer irrigation applied. Populations will be tested against the termination criteria (maximum number of iterations allowed). If the termination criteria are not satisfied, the population evolves and is re-evaluated again. The process is repeated until the termination criterion is satisfied and then the local Pareto front of the selected day combination is stored. After each iteration of the UOP, the new local Pareto is combined with the global Pareto frontier. In the next step, if there are any remaining day combinations, the above process is repeated for each new day combination until all generated random day combinations have been processed.

*Lower-level screening* Firstly, the K-means method is used to screen the global Pareto solutions with higher yield. Then, secondary screening takes economic efficiency as the objective and optimizes it by Differential Evolution (DE) algorithm. Finally, the locally appropriate solution is intelligently selected.

### Optimization strategies and configuration

Due to the complexity of the problem, a BSBOP framework was proposed in this study. Due to a large number of variables behind irrigation and fertilization, traversal date for optimization appears to be particularly difficult and time-consuming, assuming that only irrigation is optimized for 120 days of the growth cycle and the decision-maker has 0-150 mm of water per day, then there are ${151}^{120}$ different solutions. If both irrigation and fertilization are considered, then there are ${151}^{120}\cdot {151}^{120}$ different solutions. Therefore, this study tries to reduce the number of variables while minimizing the running time of the algorithm.

Here we hypothesize that more precision and effective agricultural management can be implemented through the proposed framework. Not only can crop yields be increased, but also irrigation application and fertilizer application can be reduced, while the solutions obtained have important guidance for decision-makers: such as the selection of irrigation and fertilizer application dates during the growing season of the crop, the selection of irrigation and fertilizer application amounts, and the relationship between economic benefits and application costs. To test this hypothesis, different optimization strategies were developed and evaluated (Table 1). Each optimization strategy was aimed at maximizing yield while minimizing resource wastage.

The various strategies are listed below (Table 1). Strategy 1—Fixed irrigation dates: Keeping the number of irrigation days and all parameters constant, only the amount of irrigation on each date is changed, trying to reduce the amount of irrigation as much as possible, make it easy to compare the results with best practices. Strategy 2—Optimal irrigation dates: Traverse through the irrigation dates to optimize irrigation, and try to find a better combination of irrigation dates (optimal dates) and better amount of irrigation over the wheat growth cycle. Strategy 3—Optimal irrigation dates based on surrogate model: RBF is added to Strategy 2, which makes it possible to reduce lots of time. Strategy 4—Fixed fertilizer application date: Using the optimal irrigation date found in Strategy 2 while keeping the number of days of fertilization and all other parameters constant, irrigation and fertilization are optimized in an attempt to minimize the amount of irrigation and fertilizer applied. Strategy 5—Optimal fertilizer application date: while ensuring the optimal irrigation date, traverse the fertilizer application date for optimization, trying to find out the potential yield of the crop. Strategy 6—Optimal fertilizer application date based on surrogate model: RBF is introduced based on Strategy 5. The time consumption was reduced.

The stopping criterion in this study is when the optimization results converge visually. The algorithm population size was set to 105, and the generation of offspring used traditional polynomial Mutation. The number of hidden layers of the surrogate model is equal to the dimension of the decision variables, the learning rate is 0.01, the Gaussian kernel function is chosen as the activation function of the hidden layer in the RBF network. The neurons centers are generated by the K-means clustering method. The width parameter of the function is generated by calculating the variance of each cluster. The optimization weight parameters are selected by the recursive least square method. This is because the use of the least square method is likely to encounter situations where matrix inversion is troublesome. Therefore, recursive least squares (RLS) is often used to give a recursive form of the matrix in which the inverse needs to be found, making it computationally easier.

## Results

### Fixed irrigation dates to optimize yield and amount of irrigation

#### Strategy 1—fixed irrigation dates

Strategy 1 applied the actual irrigation dates to optimize the yield and irrigation. The final optimization results indicated that the irrigation after performing the optimization was much less than the actual irrigation, and the solution with the highest yield had almost the same total yield as the best practice solution, but required 44.2% less irrigation (Table 2).

**Table 2:** Comparison of best simulated irrigation experimental results (ranked by yield) and the best practices.

Yield(kg/ha)		Total Irrigation(mm)		Reduction in irrigation usage
Best practices	Optimized results	Best practices	EMO results	
7410	7410	360	299	17%
	7399		242	32.8%
	7388		201	44.2%

In this experiment, 105 strategies were obtained by running 200 times. Obviously, not all of the strategies were high-yield solutions. For example, when the irrigation amount is 20 mm, the yield is 3846 kg/ha. Although the yield is significantly lower, it cannot be said that this solution is necessarily poor. Because when the decision-maker selects the strategy, if the local water resources are scarce, then it can only be as much as possible to ensure the yield with limited irrigation. This solution helps the decision-maker to understand the local agricultural management strategy deeply.

**Figure 4:**
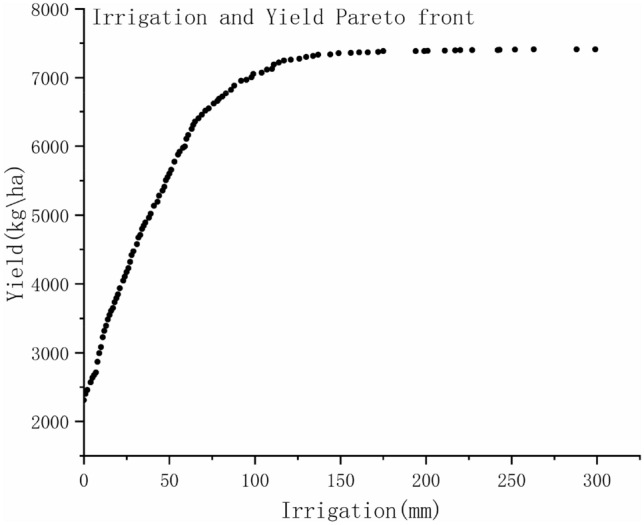
Applied the actual irrigation date to optimize yield and irrigation.

From the Fig. 4, it can be seen that the yield increases more and more slowly with a gradual increase in irrigation. So when the decision maker choose strategies, they may not simply look at the highest yield, but choose a compromise option. For example, if the choice is between Option 1 and Option 2, where Option 1 has 201 mm of irrigation and a yield of 7399 kg/ha and Option 2 has 299 mm of irrigation and a yield of 7410 kg/ha, it is clear which option the decision maker is going to choose. Option 1 reduces irrigation by almost 33% compared to Option 2, but yield do not increase much. Thus, Option 2 is not a suitable option for many stakeholders and, of course, the final choice of option is related to local conditions.

### Irrigation and yield optimization for traversal date combinations

#### Strategy 2—optimal irrigation dates

**Figure 5:**
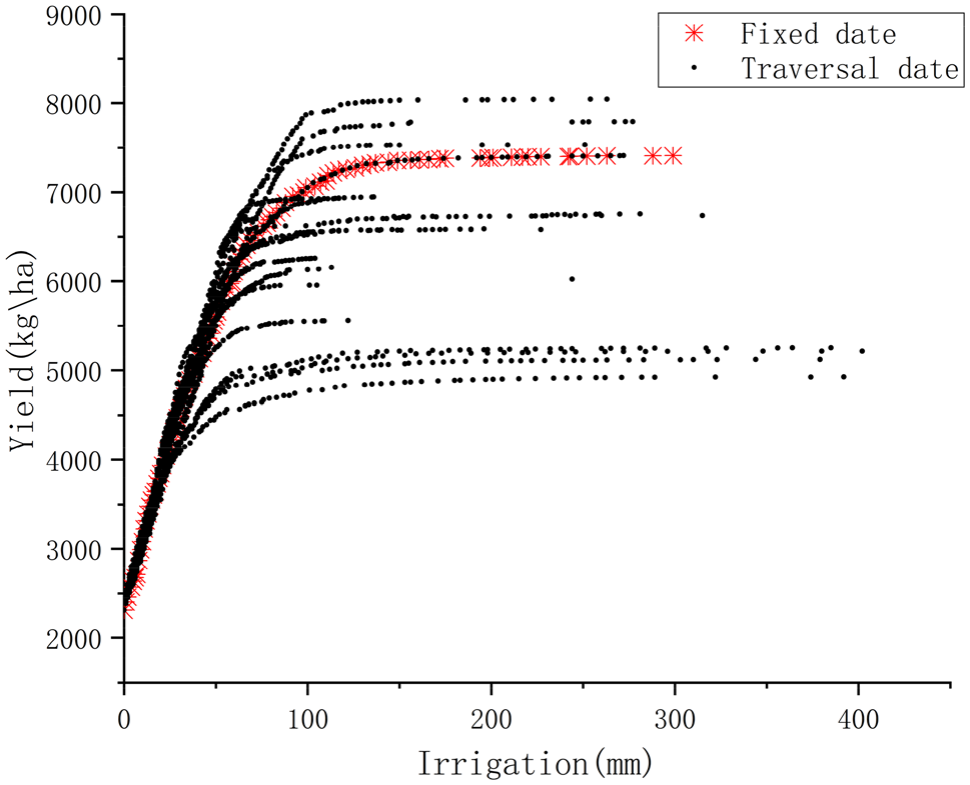
Optimization of yield and irrigation by traversal date.

The date combinations generated by the LHS, were traversed on the basis of Strategy 1. Figure 5 shows that the traversal dates were optimized for irrigation and yield, and the date combinations with higher yield than Strategy 1 were found. For example, irrigation of 201 mm in Strategy 1 resulted in a yield of 7388 kg/ha and irrigation of 263 mm in Strategy 2 resulted in a yield of 8043 kg/ha. This means that by traversing the dates we found better irrigation dates. And by the Fig. 5 it was found that irrigation had a strong contribution to the increase in crop yield. The reason for this result may be that in northwestern China, where there is a perennial drought, the crop has a very urgent need for water, which may not happen in the southern region where there is abundant rainfall.

From the Fig. 5 it can be seen that the Pareto front consisting of lots of Pareto optimal solutions of Strategy 2 outperform the Strategy 1. It is to be noted that all solutions in Strategy 1 are dominated by the solutions in Strategy 2. This result was due to the fact that we only irrigated 3 times during the planting period of the crop, which resulted in a larger effect of different date combinations on the yield.

#### Strategy 3—optimal irrigation dates based on surrogate model

**Figure 6:**
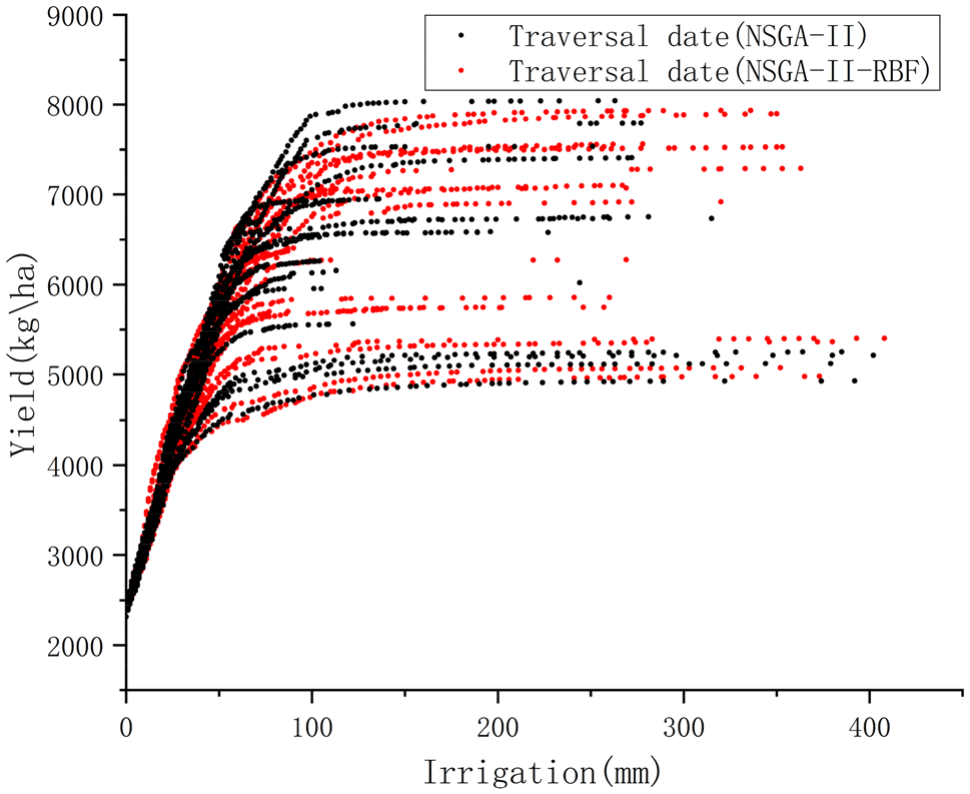
Comparison of traditional optimization algorithm for traversing irrigation dates and application of surrogate optimization for traversing irrigation dates.

Strategy 3 optimizes the yield and irrigation by RBF based on the Strategy 2. It is to be noted that the constant combinations of dates were chosen as in Strategy 2. As can be seen from the Fig. 6, there is almost no difference between the results of Strategy 2 and Strategy 3, but the time consumption is significantly reduced. The advantage of applying RBF was seen in Strategy 3.

From Table 4, the time used for the Strategy 2 is 40,523 s, while the time used after using RBF is 26,271 s. Time consumption reduces 35%, almost four hours. Imagine if we select 100 date combinations, especially in the case of limited computational resources, the time saved by applying RBF is very significant.

### Application of optimal date combinations for optimal fertilization

#### Strategy 4—fixed fertilizer application date

**Figure 7:**
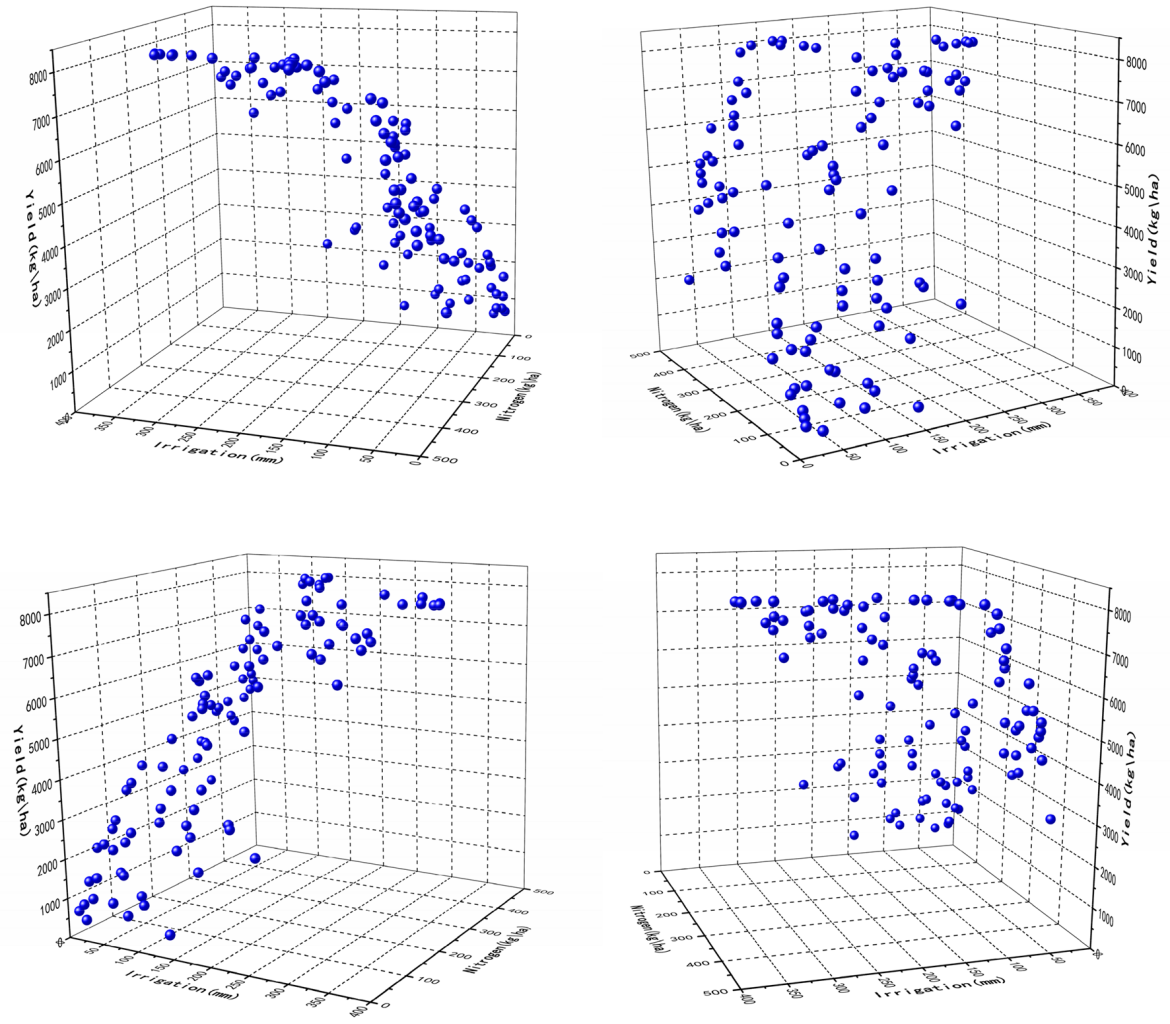
Optimization of yield, irrigation, and nitrogen application by traversing fertilizer application dates.

Strategy 4 applied the optimal date from Strategy 2 to optimize both irrigation and fertilization. The results of the optimization indicated that a similar reduction in irrigation was achieved as in Strategy 1. Fertilizer application of nitrogen was reduced by 34% (Table 3). The worst solution with 0 mm irrigation and 30 kg/ha nitrogen fertilizer applications resulted in a final yield of 953 kg/ha (Fig. 7). The worst case of Strategy 4 performed much worse in terms of yield opposed to the worst yield of Strategy 1. This is due to the fact that the amount of nitrogen fertilizer applied in Strategy 1 is fixed.

**Table 3:** Comparison of best simulated irrigation and nitrogen experimental results (ranked by yield) and the best practices.

Yield(kg/ha)		Total irrigation(mm)		Reduction in irrigation usage (%)	Total nitrogen(kg/ha)		Nitrogen reduced (%)
Best practices	Optimized results	Best practices	EMO results		Best practices	EMO results	
7410	7867	360	230	36.1	336	243	28
	7819		270	26		185	45
	7762		205	44		212	37

The low-yield solutions obtained by optimizing irrigation and nitrogen application still provide valuable agricultural insights despite the presence of lower-yield solutions in the experimental results. The solution with the lowest nitrogen application applied 0 kg/ha of nitrogen and 44 mm of irrigation and yielded 1239 kg/ha. Both solutions with the lowest irrigation applied only 11 mm of irrigation and applied 145 kg/ha and 332 kg/ha of nitrogen with yields of 2939 kg/ha and 3295 kg/ha (Fig. 7), respectively. Both of these solutions are provided to farmers in severe situations with effective management practices. Assuming a farmer wants to sell organic wheat, nitrogen fertilizer may be not used. Solutions which require little to no irrigation may be chosen in areas with strict irrigation restrictions. In both cases, the Pareto front provides the optimal yield under such constraints. Furthermore, it was found through the results that when irrigation was severely restricted, the final yield increase was limited even when the nitrogen application was greatly increased, suggesting a strong mutual promotion between water and nitrogen.

Containing the highest irrigation and nitrogen fertilizer application also provided a worthwhile trade-off solution. The solution with the highest nitrogen fertilizer application applied 426 kg/ha more nitrogen than the actual application (336 kg/ha) and yielded 8118 kg/ha. In particular, this solution compensated for its high nitrogen use by reducing irrigation. Thus, stakeholders can choose management practices that use relatively large amounts of nitrogen fertilizer, thus saving significant amounts of irrigation. Similarly, the solution with the highest irrigation use used 307 mm of irrigation and 177 kg/ha of nitrogen fertilizer for a yield of 8055 kg/ha (Fig. 7). Compared to the solution in Strategy 1, this solution in Strategy 4 yielded 8078 kg/ha with 270 mm of irrigation, reducing nitrogen application by 49%, but consuming a large amount of water. These cases provide unique solutions for unique scenarios and allow decision-makers to ultimately decide whether or not to sacrifice one objective to improve the others.

In this experiment, it was found that the amount of irrigation and the amount of nitrogen fertilizer applied directly affected the yield. In fact, this is due to the lack of fertility of the soil in the northwest and the dry weather, so the water and fertility required for crop growth are in a state of deficiency, which leads to an intuitive effect of irrigation and nitrogen fertilization on yield.

### Optimization by applying the best combination of irrigation dates to traverse fertilization dates

#### Strategy 5—optimal fertilizer application date

**Figure 8:**
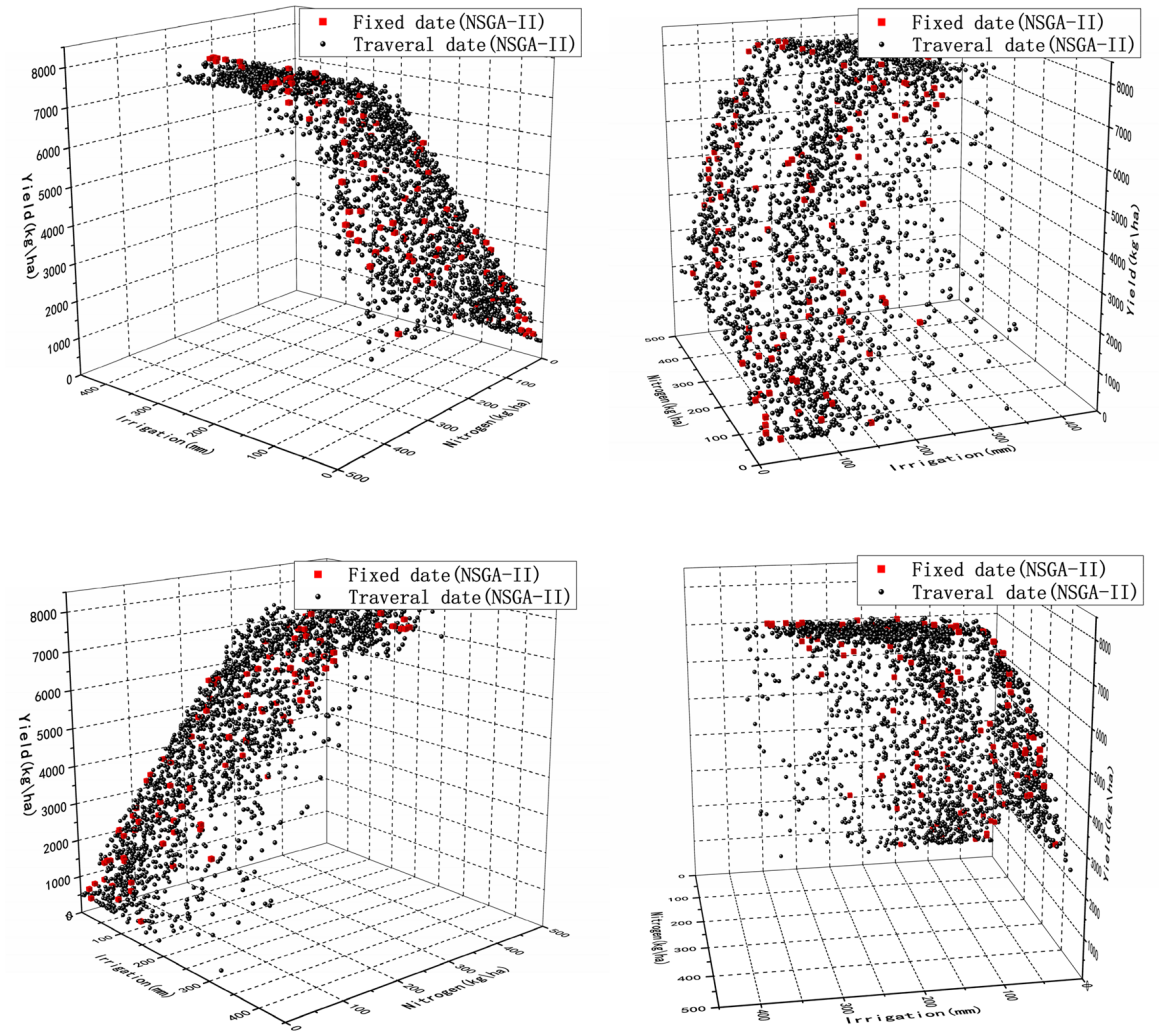
Optimization of yield, irrigation, and nitrogen application by traversing fertilizer application dates.

Strategy 5 traverses the fertilization date to optimize both fertilization and irrigation (Fig. 8 Optimization of yield, irrigation, and nitrogen application by traversing fertilizer application dates), and after optimization a better Pareto front than Strategy 4 is found. The number of solutions increases dramatically after traversing the fertilizer application dates. After 180 iterations, lots of solutions were obtained, which could provide more accurate decisions to decision-makers.

The comparison with Strategy 2 reveals an interesting phenomenon, as the irrigation date changes, the final result of Strategy 2 does not converge to the same Pareto front, but the traversal fertilization date (Strategy 5) eventually converges to the same Pareto front. In Strategy 3, it was found that fertilization and irrigation directly affect the amount of yield. Comparing strategies 2 and 3, it is concluded that irrigation appears to outperform the effect of nitrogen fertilization when crops are grown locally. This makes the trade-off between fertilizer application and irrigation clear for decision-makers. For example, in northern wheat growing areas, some farmers blindly increase the amount of nitrogen fertilizer applied in pursuit of high yield, which will not only reduce economic returns but also cause environmental pollution, so understanding the contribution of local irrigation and nitrogen fertilizer application is important to farmers.

#### Strategy 6—optimal fertilizer application date based on surrogate model

**Figure 9:**
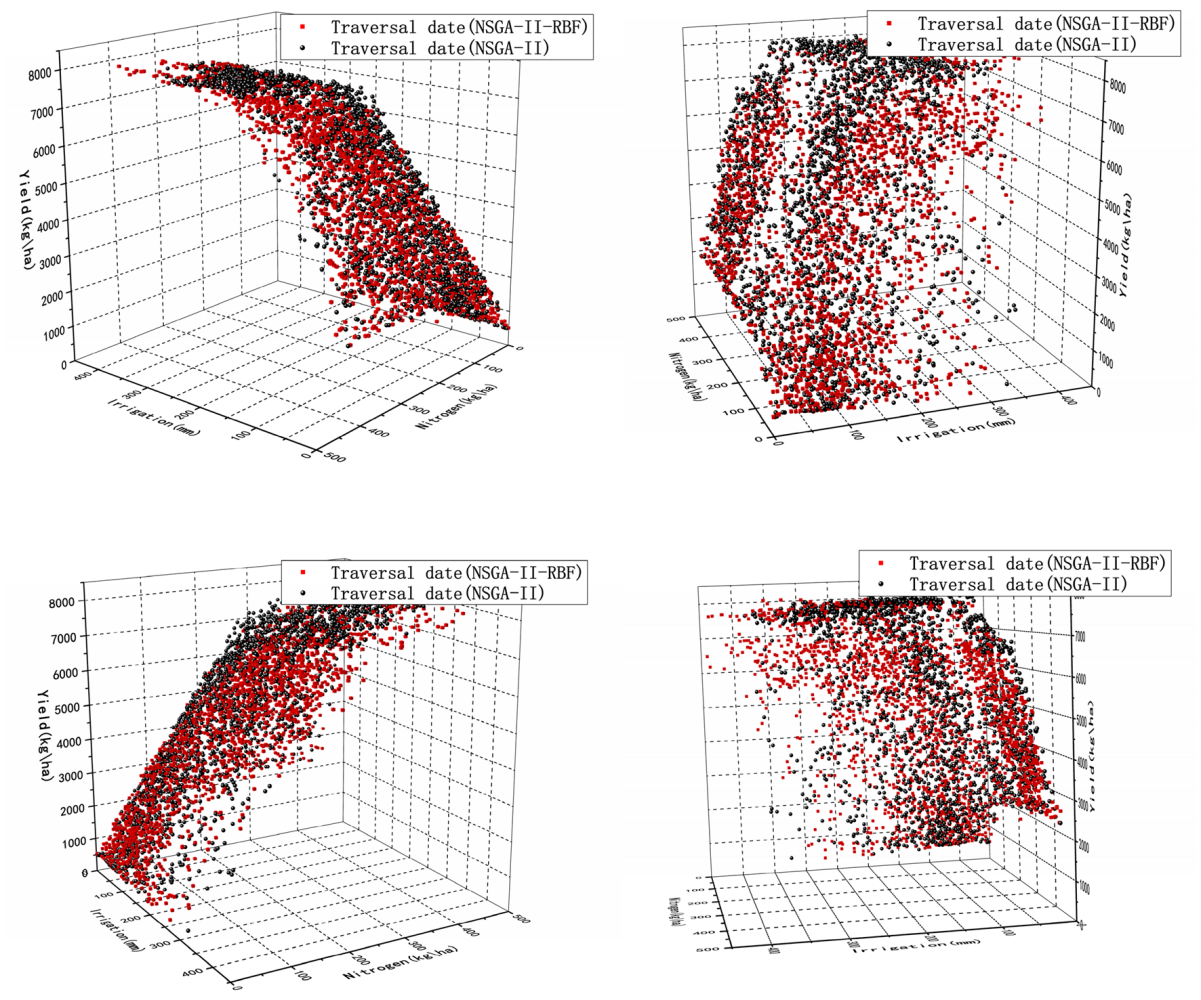
Comparison of traditional methods for optimizing traversal fertilization dates and using RBF optimization of traversal fertilization dates.

Strategy 6 introduces RBF based on Strategy 5, and the optimization process uses fewer iterations. By comparing with the image of Strategy 5 (Fig. 9), there is almost no difference between the optimized solution of Strategy 6 and the solution of Strategy 5. The time is reduced from the original 39,702 s to 23,280 s (Table 4). The time consumption is significantly reduced. This algorithm is actually trading space for time by evaluating a large number of trial offspring by RBF, which disguisedly expands the search space, and the increase of the search space will inevitably lead to less time to find the optimal solution. In particular, because we are using RBF to evaluate trial offspring. So the importance of a reliable surrogate model for the algorithm is obvious.

**Table 4:** Comparison of time consumption between applying the traditional NSGA–II and introducing the RBF surrogate model.

Strategies	Traditional NSGA–II(s)	Added RBF surrogate model to NSGA–II(s)
Strategy 2 and Strategy 3	40,523	26,271
Strategy 5 and Strategy 6	39,702	23,280

### Overall analysis and final strategy selection

**Figure 10:**
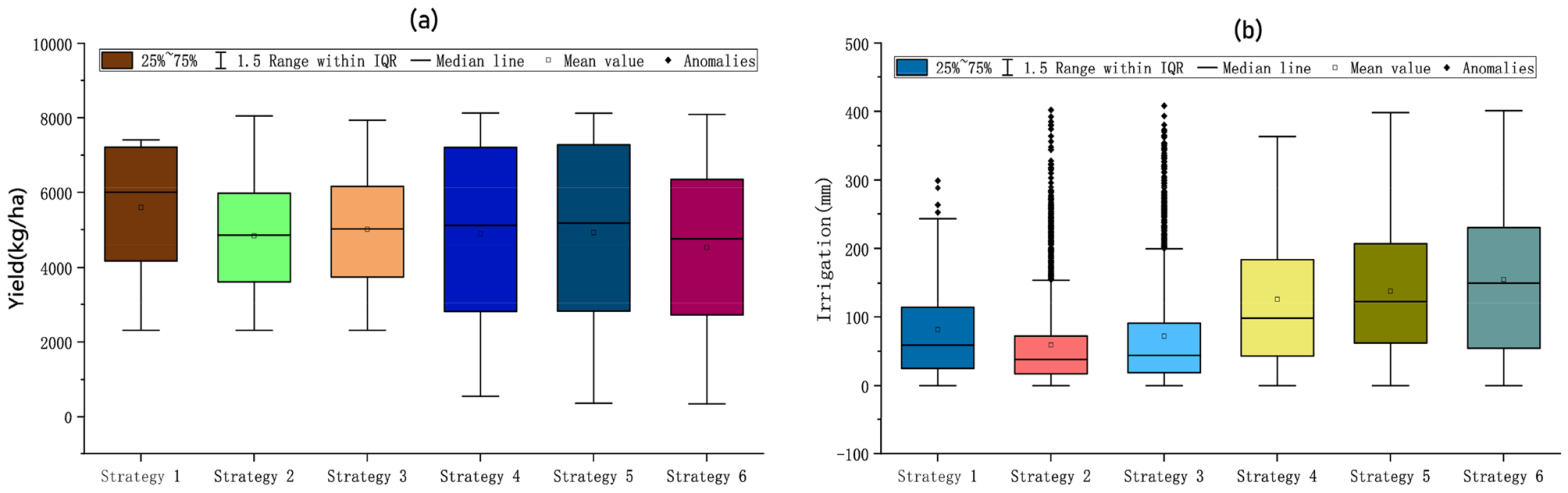
Boxplot of total irrigation(**b**) and total yield(**a**) for the six strategies.

The boxplot in Fig. 10a depicts the yield (a) and irrigation volume (b) for each of the 6 strategies. It is clear that the yield of Strategy 1 is lower than the remaining 4 strategies due to the fact that strategies 2–3 traverses the dates to find a better combination of irrigation dates, resulting in a higher yield than the actual yield. Strategy 4 also ends up with a higher yield than the actual yield because it uses the best combination of dates generated by Strategy 2. In addition, the yield minima of the first three strategies were much larger than the yield minima of the last three strategies due to the fact that we used the best fertilization strategy when performing the irrigation optimization. It is to be noted that there are anomalies in the irrigation volume for the first three strategies (Fig. 10b), which are due to the fixed amount of fertilizer applied when performing irrigation optimization, resulting in incomplete water and fertilizer effects. This also verified, in reverse, the reason for the larger yield minima for the first three strategies. Finally, as all the solutions generated by the six strategies are analyzed, this is of great importance for decision-makers to understand the local crop growth and management strategies.

**Figure 11:**
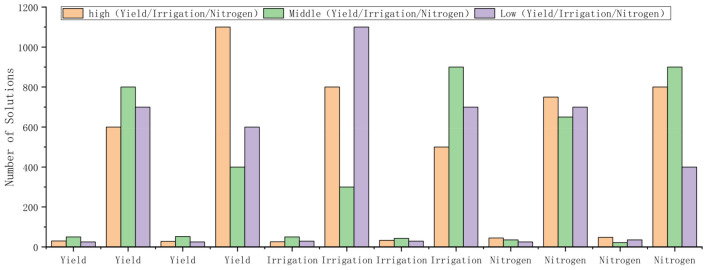
Applies the K-means method to cluster yield, irrigation and fertilizer application for strategies 1, 2, 4 and 5.

The histogram in Fig. 11 applies the k-means method to cluster yield, irrigation and fertilizer application for strategies 1, 2, 4 and 5, and classifies a large number of strategies as high (yield/irrigation/fertilizer application), medium (yield/irrigation/fertilizer application) and low (yield/irrigation/fertilizer application). According to the survey, it was found that the local water resources are relatively abundant and the price of nitrogen fertilizer is reasonable, so the choice of high yield strategy is inevitable for the local decision-makers. Such choices would have the disadvantage that high yield does not mean that the highest economic benefit can be obtained, so we investigated the local fertilizer, irrigation costs and labor costs, treated all high yield strategies as initial populations, and optimized them by using a single objective algorithm (DE) to find the strategy that produces the highest economic benefit.

The field survey revealed that the cost required for irrigation has not changed and the cost of fertilizer application has fluctuated slightly over the past few years (Table 5), but the price of wheat has continued to decline. In other words, farmers' income decreased over time, and the optimization results for DE showed that the net profit of wheat increased by almost 8% after optimization (Table 6), which is very valuable for stakeholders. It was screened that numerous strategies by the profitability issue that is of most interest to stakeholders. This makes the final strategy more reliable and reduces the decision maker's time to choose a strategy.

**Table 5:** The price of wheat in recent years and the cost of irrigation and fertilization.

Year	Wheat price(yuan/kg)	Irrigation costs((yuan·mm)/ha)	Fertilization costs(yuan/kg)
2017	2.36	2.14	1.62
2018	2.3	2.14	1.78
2019	2.24	2.14	1.72
2020	2.24	2.14	1.86

**Table 6:** Comparison of best simulated experimental results (sorted by net profit) and the best practices.

Yield(kg/ha)		Net profit(kg/ha)		Increased economic benefits (%)
Best practices	Optimized results	Best practices	EMO results	
7410	8118	16,428	17,847	8
	8102		17,833	7.9
	8070		17,821	7.8

## Discussion

A limitation to this paper is that, as with most evolutionary algorithms, there is no theoretical guarantee that the use of a surrogate-assisted model will converge to the Pareto front. Moreover, the approximation may be inaccurate due to the computational cost of the simulation that generates the objective. It is worth noting that the convergence of the obtained algorithm and the diversity of Pareto non-dominated solutions depend on the quality of the approximation of the target surrogate model. If the model is inaccurate, the obtained Pareto non-dominated solutions may be far from the actual Pareto front or may miss some regions of the Pareto front. However, since only one surrogate is used in this study, the correction and maintenance of the surrogate will be relatively simple, and since the RBF model (with only one hidden layer) is used, the parameters of the Gaussian kernel function are also accurately calculated, which makes the model much more stable and predictive. Therefore, in this paper, the quality of the model is guaranteed.

Wheat is the second-largest grain crop in Shaanxi Province. Local farmers care more about the economic benefits of the crop than the yield. In addition, the large number of solutions generated by the NSGA–II algorithm is not necessarily adapted to the local environment. Since the local weather and soil environment are not likely to change much in the next few years. Therefore, by focusing on the economic benefits that farmers are most concerned about, firstly, we select the solutions that are suitable for the local area through K-means clustering, and then perform a lower-level screening of a large number of solutions by calculating the net profit of the crops, we can find the high-yield solutions that are suitable for the local area. Solutions are reduced from thousands to single-digit size. This not only reduces the time spent on selecting solutions for decision-makers but also makes it more intuitive for decision-makers to get locally adapted solutions.

## Conclusion

Although many algorithms combining MOP and agricultural simulation models have been proposed, a BSBOP framework, which combines NSGA-II, RBF and DSSAT, were not reported, which is a significant contribution in this regard. Secondly, the K-means^51^ method was used to cluster the large number of solutions generated by different strategies into solutions with high yield, low irrigation and low fertilizer application, and then the solutions suitable for the local area are selected, followed by economic analysis. Finally, the solutions were evaluated to find the economically suitable solution for the local area, which is friendly to decision-makers because the most important concern of crop-growing decision-makers in a region is the issue of economic efficiency, and by reducing the number of solutions, the difficulty of decision-makers in selecting solutions is greatly reduced.

The proposed approach applied NSGA-II and RBF to the DSSAT model to generate multiple approximate Pareto solutions, which can be presented to the decision-maker and can then be used to analyze the optimization of trade-offs among various objectives. As with other multi-objective optimization algorithms, surrogate-assisted models are very flexible and allow us to simulate not only the objective function but also works well for constrained problems. This is typical in many simulation-based problems. More importantly, it is very meaningful for applying computationally expensive models of DSSAT by using a combination of surrogate-assisted models and multi-objective optimization algorithms. In addition to its application to agricultural models, it can also apply to engineering design optimization and EDM parameter optimization.

Agricultural management often requires simultaneous optimization of multiple objectives. The application of multi-objective optimization algorithms allows to obtain a large number of optimal solutions, which can provide decision-makers with the best solution available. In addition, due to the expensive objective function, the RBF surrogate model is applied to reduce time consumption. Since we propose a bi-level screening and bi-level optimization framework that is more in line with the laws of agricultural management, it not only reduces the running pressure of the program but also allows intelligent selection of the strategy that satisfies the decision-maker. In the case of agriculture, in addition to economic benefits, we can also consider the impact of environmental and socio-economic factors on agricultural management that play an important role in the management of crops for sustainable agricultural intensification.

## Supplementary Information

Supplementary Information.

## Data Availability

The raw data, code and experimental results used in the paper are in the supplementary file. Please see supplementary materials for more details.

## References

[1] Mueller ND (2012). Closing yield gaps through nutrient and water management. Nature.

[2] Pradhan P, Fischer G, van Velthuizen H, Reusser DE, Kropp JP (2015). Closing yield gaps: How sustainable can we be?. PLoS ONE.

[3] Parsinejad M, Yazdi AB, Araghinejad S, Nejadhashemi AP, Tabrizi MS (2013). Optimal water allocation in irrigation networks based on real time climatic data. Agric. Water Manag..

[4] Zhang, B., Wang, Y., Wu, C., Liu, H. & Li, J. In *2021 9th International Conference on Agro-Geoinformatics (Agro-Geoinformatics).* 1–6.

[5] Kropp I (2019). A multi-objective approach to water and nutrient efficiency for sustainable agricultural intensification. Agric. Syst..

[6] Shi, Q., Wang, Y. & Li, Y. In *2021 9th International Conference on Agro-Geoinformatics (Agro-Geoinformatics).* 1–5.

[7] Jones JW (2003). The DSSAT cropping system model. Eur. J. Agron..

[8] D.M., B. CERES-Maize: A simulation model of maize growth and development: Jones, C.A. and Kiniry, J.R. (Editors), Texas A & M University Press, College Station, TX, 1986, 194 pp. + two diskettes. U.S. $33.50 (cloth). *J. Agric. Forest Meteorol*. **41** (1987).

[9] Boote K, Jones J (1998). Simulation of crop growth: Cropgro model. Agric. Syst. Modeli. Simulat..

[10] Zhou YW, Fan HH (2018). Research on multi objective optimization model of sustainable agriculture industrial structure based on genetic algorithm. J. Intell. Fuzzy Syst..

[11] Ragkos, A. & Psychoudakis, A. Minimizing adverse environmental effects of agriculture: A multi-objective programming approach. *J. Oper. Res.* **9** (2009).

[12] Sarker, R. & Ray, T. An improved evolutionary algorithm for solving multi-objective crop planning models. J. Comput. Electron. Agric. **68** (2009).

[13] Han J, Hu Y, Mao M, Wan S (2020). A multi-objective districting problem applied to agricultural machinery maintenance service network. Eur. J. Oper. Res..

[14] Deb, K., Pratap, A., Agarwal, S. & Meyarivan, T. J. I. t. o. e. c. A fast and elitist multiobjective genetic algorithm: NSGA-II. **6**, 182–197 (2002).

[15] Corne, D. W., Jerram, N. R., Knowles, J. D. & Oates, M. J. In *Proceedings of the 3rd Annual Conference on Genetic and Evolutionary Computation.* 283–290.

[16] Zhang, Q. & Li, H. J. I. T. O. E. C. MOEA/D: A multiobjective evolutionary algorithm based on decomposition. **11**, 712–731 (2007).

[17] Zitzler, E., Laumanns, M. & Thiele, L. J. T.-r. SPEA2: Improving the strength Pareto evolutionary algorithm. **103** (2001).

[18] Zhou, A. *et al.* Multiobjective evolutionary algorithms: A survey of the state of the art. **1**, 32-49 (2011).

[19] Jin, Y. J. S. & Computation, E. Surrogate-assisted evolutionary computation: Recent advances and future challenges.**1**, 61-70 (2011).

[20] Fleming, P. J. & Purshouse, R. C. J. C. E. P. Evolutionary algorithms in control systems engineering: A survey. **10**, 1223–1241 (2002).

[21] Chugh, T., Kratky, T., Miettinen, K., Jin, Y. & Makonen, P. In *Proceedings of the Genetic and Evolutionary Computation Conference.* 1147–1155.

[22] Jin, Y. & Sendhoff, B. J. I. C. I. M. A systems approach to evolutionary multiobjective structural optimization and beyond. **4**, 62-76 (2009)

[23] Jin Y, Wang H, Chugh T, Guo D, Miettinen K (2019). Data-driven evolutionary optimization: An overview and case studies. IEEE Trans. Evol. Comput..

[24] Chen, G., Luo, X., Jiao, J. J. & Xue, X. Data-driven evolutionary algorithm for oil reservoir well-placement and control optimization. *Fuel* **326**, 125125, doi:10.1016/j.fuel.2022.125125 (2022).

[25] Long H, Li P, Gu W (2020). A data-driven evolutionary algorithm for wind farm layout optimization. Energy.

[26] Belhaiza, S., M’Hallah, R., BenBrahim, G. & Laporte, G. (2019) Three multi-start data-driven evolutionary heuristics for the vehicle routing problem with multiple time windows. *J. Heurist.* **25**, 485–515, 10.1007/s10732-019-09412-1.

[27] Morala P, Cifuentes JA, Lillo RE, Ucar I (2021). Towards a mathematical framework to inform neural network modelling via polynomial regression. Neural Netw..

[28] Kang, Q., Liao, W.-k., Agrawal, A. & Choudhary, A. In *Proceedings of the 25th ACM International on Conference on Information and Knowledge Management* 2209–2214 (Association for Computing Machinery, Indianapolis, Indiana, USA, 2016).

[29] Johnson MJ (2004). An error analysis for radial basis function interpolation. Numer. Math..

[30] Rocha H, Dias JM (2019). Early prediction of durum wheat yield in Spain using radial basis functions interpolation models based on agroclimatic data. Comput. Electron. Agric..

[31] Lawrence T, Zhang L, Lim CP, Phillips EJ (2021). Particle swarm optimization for automatically evolving convolutional neural networks for image classification. IEEE Access.

[32] Nguyen, T.-S. *High Performance Neural Networks for Online Speech Recognizer*, (2021).

[33] Achieng KO (2019). Modelling of soil moisture retention curve using machine learning techniques: Artificial and deep neural networks vs support vector regression models. Comput. Geosci.-Uk.

[34] de Oliveira MA, Possamai O, Dalla Valentina LVO, Flesch CA (2013). Modeling the leadership–project performance relation: Radial basis function, Gaussian and Kriging methods as alternatives to linear regression. Expert Syst. Appl..

[35] Xie T (2018). Advanced multi-objective robust optimization under interval uncertainty using kriging model and support vector machine. J. Comput. Inform. Sci. Eng..

[36] Kropp, I. *et al.* A multi-objective approach to water and nutrient efficiency for sustainable agricultural intensification %J Agricultural Systems. **173** (2019).

[37] Jones, C., Kiniry, J. J. T. A. & M University Press, C. S. A simulation model of maize growth and development. (1986).

[38] Ritchie, J. J. A. W. Y. P. Description and performance of CERES wheat: A user-oriented wheat yield model. 159–175 (1985).

[39] Boote, K. J., Jones, J. W. & Hoogenboom, G. In *Agricultural Systems modeting and Simulation* 651–692 (CRC Press, 2018).

[40] Porter CH (2014). Erratum to: Modeling organic carbon and carbon-mediated soil processes in DSSAT v4. 5. Oper. Res..

[41] Jiang R (2019). Exploring management strategies to improve maize yield and nitrogen use efficiency in northeast China using the DNDC and DSSAT models. Comput. Electron. Agric..

[42] Rivas-Dávalos, F. & Irving, M. R. In *International Conference on Evolutionary Multi-Criterion Optimization.* 707–720 (Springer).

[43] Jia, L., Wang, Y. & Fan, L. J. I. C.-A. E. *Multiobjective bilevel optimization for production-distribution planning problems using hybrid genetic algorithm*. 21, 77-90 (2014).

[44] Pires, E. J. S., de Moura Oliveira, P. B. & Machado, J. A. T. In *Workshops on Applications of Evolutionary Computation.* 219–229 (Springer).

[45] Deb K, Pratap A, Agarwal S, Meyarivan T (2002). A fast and elitist multiobjective genetic algorithm: NSGA-II. IEEE Trans. Evol. Comput..

[46] Jin, Y. J. S. c. A comprehensive survey of fitness approximation in evolutionary computation. **9**, 3–12 (2005).

[47] Wang, H., Jin, Y., Sun, C. & Doherty, J. J. I. T. o. E. C. Offline data-driven evolutionary optimization using selective surrogate ensembles. **23**, 203–216 (2018).

[48] Sun, C., Zeng, J., Pan, J., Xue, S. & Jin, Y. J. I. s. A new fitness estimation strategy for particle swarm optimization. **221**, 355–370 (2013).

[49] Liu, B., Zhang, Q. & Gielen, G. G. J. I. T. o. E. C. A Gaussian process surrogate model assisted evolutionary algorithm for medium scale expensive optimization problems. **18**, 180–192 (2013).

[50] Datta R, Regis RG (2016). A surrogate-assisted evolution strategy for constrained multi-objective optimization. Expert Syst. Appl..

[51] Hofmeyr DP (2020). Degrees of freedom and model selection for *k*-means clustering. Comput. Stat. Data Anal..

